# Report on ISCTM Consensus Meeting on Clinical Assessment of Response to Treatment of Cognitive Impairment in Schizophrenia

**DOI:** 10.1093/schbul/sbv111

**Published:** 2015-09-11

**Authors:** Richard S. E. Keefe, George M. Haig, Stephen R. Marder, Philip D. Harvey, Eduardo Dunayevich, Alice Medalia, Michael Davidson, Ilise Lombardo, Christopher R. Bowie, Robert W. Buchanan, Dragana Bugarski-Kirola, William T. Carpenter, John T. Csernansky, Pedro L. Dago, Dante M. Durand, Frederick J. Frese, Donald C. Goff, James M. Gold, Christine I. Hooker, Alex Kopelowicz, Antony Loebel, Susan R. McGurk, Lewis A. Opler, Amy E. Pinkham, Robert G. Stern

**Affiliations:** ^1^Department of Psychiatry, Duke University Medical Center, Durham, NC;; ^2^Department of Neuroscience Clinical Development, Abbvie, North Chicago, IL;; ^3^Semel Institute for Neuroscience at UCLA, and VA Desert Pacific Mental Illness Research, Education, and Clinical Center, Los Angeles, CA ;; ^4^Department of Psychiatry and Behavioral Sciences, University of Miami School of Medicine, Miami, FL;; ^5^Clinical Development, Takeda, Los Angeles, CA;; ^6^Department of Psychiatry, Columbia University, New York, NY;; ^7^Department of Psychiatry, The Chaim Sheba Medical Center, Tel Hashomer, Israel;; ^8^Clinical Research, FORUM Pharmaceuticals, Waltham, MA;; ^9^Department of Psychology, Queen’s University, Kingston, Ontario, Canada;; ^10^Department of Psychiatry, Maryland Psychiatric Research Center, University of Maryland School of Medicine, Baltimore, MD;; ^11^Department of Psychiatry, F. Hoffmann-La Roche Ltd., Basel, Switzerland;; ^12^Department of Psychiatry, Northwestern University Feinberg School of Medicine, Evanston, IL;; ^13^Department of Psychiatry and Behavioral Sciences, University of Miami School of Medicine, Miami, FL;; ^14^Department of Psychiatry, Northeast Ohio Medical University, Rootstown, OH;; ^15^Department of Psychiatry, Nathan Kline Institute, and New York University School of Medicine New York, NY;; ^16^Harvard University;; ^17^Psychiatry and Behavioral Sciences, David Geffen School of Medicine at UCLA, Los Angeles, CA;; ^18^Global Clinical Development, Sunovion, Ft. Lee, NJ;; ^19^Center for Psychiatric Rehabilitation, Boston University, Boston, MA;; ^20^School of Behavioral and Brain Sciences, The University of Texas at Dallas, Richardson, TX;; ^21^Essex County Hospital Center, Cedar Grove, NJ

**Keywords:** cognitive assessment, neuropsychology, treatment

## Abstract

If treatments for cognitive impairment are to be utilized successfully, clinicians must be able to determine whether they are effective and which patients should receive them. In order to develop consensus on these issues, the International Society for CNS Clinical Trials and Methodology (ISCTM) held a meeting of experts on March 20, 2014, in Washington, DC. Consensus was reached on several important issues. Cognitive impairment and functional disability were viewed as equally important treatment targets. The group supported the notion that sufficient data are not available to exclude patients from available treatments on the basis of age, severity of cognitive impairment, severity of positive symptoms, or the potential to benefit functionally from treatment. The group reached consensus that cognitive remediation is likely to provide substantial benefits in combination with procognitive medications, although a substantial minority believed that medications can be administered without nonpharmacological therapy. There was little consensus on the best methods for assessing cognitive change in clinical practice. Some participants supported the view that performance-based measures are essential for measurement of cognitive change; others pointed to their cost and time requirements as evidence of impracticality. Interview-based measures of cognitive and functional change were viewed as more practical, but lacking validity without informant involvement or frequent contact from clinicians. The lack of consensus on assessment methods was viewed as attributable to differences in experience and education among key stakeholders and significant gaps in available empirical data. Research on the reliability, validity, sensitivity, and practicality of competing methods will facilitate consensus.

## Introduction

Cognitive impairment is a significant contributor to disability and poor functional outcomes in patients with schizophrenia.^1,2^ While antipsychotic medications are effective at reducing the psychotic symptoms of the illness, they have little impact on cognitive symptoms,^3,4^ and there are currently no approved treatments for cognitive impairment associated with schizophrenia (CIAS). This significant unmet need has been the focus of large government and industry initiatives in the United States^5^ and Europe,^6^ which have stimulated and facilitated large drug development programs for the treatment of CIAS.^7,8^ Further, several behaviorally based cognitive remediation treatments have demonstrated modest success at improving CIAS,^9^ and Food and Drug Administration (FDA) device clearance trials are ongoing (www.clinicaltrials.gov, NCT01422902). It is likely that by the end of this decade, a pharmacological or remediation-based treatment for CIAS will be approved by FDA and/or other regulatory agencies.

Prescribing physicians have become familiar with the evaluation of psychotic symptoms such as delusions, hallucinations, and agitation, which are the target of antipsychotic medications. These symptoms are often at the forefront of the clinical evaluation of the person with schizophrenia, partially because they appear to call out for immediate intervention in order to mitigate against ongoing patient suffering and harm to others. However, despite decades of research emphasizing the importance of cognition in predicting outcome in schizophrenia and other disorders, evaluation of cognition is not a part of standard education or training, even in many advanced psychiatry residency programs and fellowships, and is not a component of a standard psychiatric diagnostic interview. If treatments for CIAS are to be utilized efficiently and successfully, it will be essential for physicians and other clinicians to be able to determine which patients should receive them, and whether such treatments are effective. The assessment of cognition in clinical practice needs development now so that appropriate tools and approaches will be available when treatments are approved for this indication. In order to generate discussion on these issues and to determine whether consensus is possible, the International Society for CNS Clinical Trials and Methodology (ISCTM) held a meeting to seek input from top experts in the field on March 20, 2014, in Washington, DC. While this group of experts was not without their own biases and potential conflicts of interest, it is a strong and effective tradition of ISCTM not to exclude those with conflicts of interest, but to require that they are transparent about their potential conflicts, and allow them to express their opinions in the open.

### Aims of Meeting

The objective of this meeting was to determine the existing level of consensus on (1) methods for monitoring response to procognitive medications and interventions for patients with schizophrenia; (2) the necessary tools and training to conduct this assessment in the clinic setting; and (3) approaches to prescribing procognitive medications and interventions in the clinic.

## Methods

### Survey

Survey questions were developed by the project Steering Committee and sent to 46 experts in schizophrenia, cognition, clinical trials, community psychiatry, and drug development. They were selected on the basis of their field of expertise, to ensure adequate representation from all areas of interest, and their availability. The group included academic psychologists and psychiatrists, community psychiatrists, physicians, pharmacologists and psychologists from industry, and a consumer representative. Thirty-four (73%) respondents completed the survey. A small number of questions were not clearly understood based on comments from the experts and data from these questions were disregarded. Most questions revealed significant disagreement or divergence of opinions and were the focus of discussion at this meeting. The data collected from the survey helped to shape the questions that would be addressed at the consensus meeting.

### Discussion

A group of 23 experts in cognition, schizophrenia, community psychiatry, and drug development were selected from the pool of 46 experts who completed the survey and invited to participate as panelists (herein referred to as “panelists”) at the consensus meeting. They were selected on the basis of their perceived contributions to this area of research and their field of expertise to ensure adequate representation from all areas of interest. The panel included 8 academic psychologists, 7 academic psychiatrists, 5 community psychiatrists, 4 physicians and a pharmacologist from industry, and a consumer representative. Fourteen panel members were prescribing physicians. In addition, the consensus meeting was open to audience participants (herein referred to as “participants”) who were interested in the discussion. The size of the audience participants was capped at 70.

The meeting began with a brief description, including pros and cons, of several cognitive assessment methods to ground panelists in their understanding of these tools. The central theme under consideration was to address the preferred method, if any, for monitoring response to treatment assuming that a medication or intervention is available for treating cognitive impairment in schizophrenia. Factors considered were complexity of administration, sensitivity and reliability, time, costs, reimbursement, and training. The remainder of the meeting consisted of very brief presentations on opposing sides of an issue or question, followed by extensive discussion by all panelists. At the end of the meeting, all of the questions that were discussed and debated during the conference were posed to the panelists for a final vote, and their responses were recorded with an audience response system. The audience participants were asked to record their responses on paper forms, which were collected following the meeting. This report is a product of the discussions and final voting at the meeting.

### Review of Assessment Methods for Cognition and Functional Outcomes

In order to establish whether a patient is responding to treatment for CIAS, there are a variety of options available. One decision is whether to focus on cognitive performance, which is the most immediate treatment target, or to focus on functional outcomes, which have greater clinical meaning and relevance to a patient’s everyday life, but may be more challenging to observe change. For the purposes of this discussion, the following categories were used: comprehensive cognitive performance assessments, brief cognitive performance assessments, interview-based measures of cognition, performance-based measures of functional capacity, and interview-based assessment of real-world functioning. These assessment methods are summarized in table 1.

**Table 1.** Cognitive Assessment Methods

Category	Examples	Advantages	Disadvantages	References
Comprehensive Cognitive Performance Assessments	• MATRICS Consensus Cognition Battery (MCCB) • CogState • Cambridge Neuropsychological Test Automated Battery (CANTAB) • Numerous tests available in all 7 MATRICS domains	• Addresses all 7 cognitive domains recognized by MATRICS • Sufficient items to generate test-retest reliability that will enable sensitivity to change • Associated with change index to calculate the amount of change that will define improvement or worsening	• Time requirements: 75min to administer; 30min to score and interpret for MCCB (less time for CogState and CANTAB) • Missing data can create scoring challenges • Require adequate tester training and credentials, as well as supervision of test administration • Many are copyrighted and have acquisition costs	Nuechterlein *et al*,^10 ^ Pietrzak *et al*,^34 ^ and Barnett *et al* ^35 ^
Brief Cognitive Performance Assessment	• Repeatable Battery for the Assessment of Neuropsychological Status (RBANS) • Brief Assessment of Cognition in Schizophrenia (BACS) • Brief Cognitive Assessment • Brief Cognitive Assessment Tool for Schizophrenia • Brief Neurocognitive Assessment (BNA)	• Addresses most domains but administered in shorter period of time than comprehensive batteries • Evidence suggests that shorter tests are equally sensitive • Lower costs	• Short tests or test batteries often have reduced reliability • Reduced number of domains tested • Testers require training and supervision	Green *et al*,^12 ^ Buchanan *et al*,^13 ^ Umbricht *et al*,^14 ^ Velligan *et al*,^22 ^ Hurford *et al*,^23 ^ and Fervaha *et al* ^24 ^
Performance-based Measures of Functional Capacity	• UCSD Performance- based Skills Assessment (UPSA)—several variants • Test of Adaptive Behavior in Schizophrenia (TABS) • Independent Living Scales (ILS)	• Able to predict failures to achieve milestones in vocational, residential, and social domains in schizophrenia and bipolar disorder populations • Functional capacity is more proximal to everyday functional deficits than cognitive impairments • Correlation between performance on functional capacity measures and cognitive tests has been remarkably consistent and substantial • Measures of functional capacity may be more strongly correlated with real-world functioning than cognitive measures • Easily tolerated and practical to utilize • Demonstrated high levels of test-retest reliability, minimal practice effects, and minimal missing data in large-scale clinical trials	• Relationship of these functional capacity measures to cognitive change may be indirect • Most are in a paper and pencil format • Comprised of several functional tasks that are not required consistently across different cultures • Most lack alternate forms which make them prone to high practice effects • Prone to ceiling effects in high functioning patients, limiting sensitivity	Green *et al*,^12 ^ Mausbach *et al*,^27 ^ Mausbach *et al*,^28 ^ Bowie *et al*,^29 ^ Leifker *et al*,^36 ^ Green *et al*,^32 ^ Keefe *et al*,^33 ^, Velligan *et al*,^37 ^ and Bowie *et al* ^38 ^
Interview-based Measures of Cognition	• Cognitive Assessment Interview (CAI) • Measure of Insight into Cognition • Schizophrenia Cognition Rating Scale (SCoRS)	• Brief administration time, requiring (~15min per interview) • Good relationship to real-world functioning • Good test-retest reliability • High correlation with some performance-based measures of cognition	• Weak relationship to objective cognitive and functional measures • Validity and correlations with performance- based measures depends upon the availability of informant • Some training is required	Green *et al*,^12 ^ Green *et al*,^32 ^ Saperstein *et al*,^39 ^ Keefe *et al*,^40 ^ and Ventura *et al*,^41 ^
Interview-based Assessments of Real-world Functioning	Specific Levels of Functioning (SLOF)	• Assesses social functioning, vocational or nonvocational productive functioning, and residential independence and self-care • Functional scales acceptably correlated with performance-based measures	• Requires input of informants such as friends or relatives or those with a caregiver relationship • Changes are likely to take far longer to detect	Durand *et al*,^42 ^ Sabbag *et al*,^46 ^ and Patterson *et al*,^42,44 ^ Buchanan *et al* ^45 ^

#### Comprehensive Cognitive Performance Assessment.

Cognition, or the ability to think and process information, has several components or domains.^10,11^ The Neurocognition Subcommittee of the Measurement and Treatment Research to Improve Cognition in Schizophrenia (MATRICS) Project chose 7 of them for the MATRICS Consensus Cognition Battery (MCCB) to be used in clinical trials assessing the efficacy of cognition-enhancing medications.^10^ Considerations for selection of domains were severity of impairment, relation to functional outcome, and feasibility for clinical trials.

Patients with schizophrenia vary greatly in their profile and severity of their cognitive impairment. Only by comprehensive assessment of various domains of cognitive functioning can response to the relevant aspects of cognitive impairment treatment be properly monitored. Further, superficial assessment of a cognitive domain is not sufficient; to be useful in clinical practice, cognitive tests require enough items to generate test-retest reliability that will enable sensitivity to change.^12–14^ Short tests or test batteries often have reduced reliability. A reliable change index calculates the amount of change that will be consistent with a predetermined percentage of change in a distribution. Usually, the chosen cutoff is 90%, meaning only 10% of the population will demonstrate that amount of change by chance. As an example, recent data collected on the MATRICS battery and its domains suggest that an approximately 10 point change on the MCCB composite score is needed to be 90% confident that the change is due to treatment effects.^15^ This is consistent with earlier studies of other batteries as well as measures of functional capacity.^16–20^ For clinicians who wish to relax the threshold for what is considered treatment response or worsening, it is possible to use a reliable change index with a 80% CI, which will reduce the MCCB composite score threshold to about 8 points.

The primary drawbacks of comprehensive assessment however are time and personnel required. The MCCB requires about 75min to administer to the patient, with scoring and interpretation requiring at least another 30min for the tester. Other comprehensive test batteries may require as much or more time. Computerized test batteries such as the CogState battery or the Cambridge Neuropsychological Test Automated Battery (CANTAB) assess most or all of the MATRICS domains. These computerized batteries may require less time for scoring but may have other implementation challenges, such as missing data.^21^ Many clinical practices have neither the time nor staff members with the proper training to complete cognitive assessment. This issue is reduced with computerized tests; however, adequate tester training and credentials, as well as supervision of test administration, are essential. Many test batteries are copyrighted and have acquisition costs that reduce enthusiasm for this method of assessment.

#### Brief Cognitive Performance Assessment.

There are test batteries available with various numbers of assessments. The Repeatable Battery for the Assessment of Neuropsychological Status (RBANS) has 10 tests covering 5 domains and requires 45min, and the Brief Assessment of Cognition in Schizophrenia (BACS) has 6 tests and requires 35min. Various combinations of existing tests have also been studied, including the Brief Cognitive Assessment,^22^ the Brief Cognitive Assessment Tool for Schizophrenia,^23^ each of which trim the number of tests to 3, and then most recently the Brief Neurocognitive Assessment (BNA) which has only 2 tests.^24^ The ideal length of a test battery for evaluating general cognitive changes in a clinical setting is not established. The amount of variance in overall test performance that is accounted for by each successive test suggests diminishing returns after about 4 tests,^25^ but some argue that smaller batteries are equally sensitive.^23,24^

The drawbacks to using brief assessments are that they reduce the number of domains that can be tested and can reduce the test-retest reliability of assessment and jeopardize the ability of the assessment to detect treatment benefit or decline. Thus, treatments with significant benefits to patients can look like failures, and deleterious effects on cognition will appear benign. The costs of brief batteries are less, but staff members who have proper credentials and supervision remain essential. Companies that sell the tests require supervision by a psychologist because subtle changes in test administration and scoring can have a tremendous effect on a patient’s scores.

#### Performance-Based Measures of Functional Capacity.

A recent development in research on the determinants of disability in schizophrenia has been performance-based measures of functional capacity.^26^ These assessments have found that impairments predict failures to achieve milestones in vocational, residential, and social domains^27,28^ in schizophrenia and bipolar disorder populations.^29,30^ Whether everyday functioning is defined either by milestone achievement^31^ or by ratings generated by high-contact informants,^29^ impairments on measures of functional capacity have typically been found to be more proximal to everyday functional deficits than cognitive impairments.^20,29^ Further, the correlation between performance on functional capacity measures and cognitive tests has been remarkably consistent and substantial, typically *r* = .60 or greater.^32,33^ Measures of functional capacity may be more strongly correlated with real-world functioning than cognitive measures.^29^

Functional capacity measures are easily tolerated and practical to utilize.^12,32,37^ They have demonstrated high levels of test-retest reliability, minimal practice effects, and minimal missing data in large-scale clinical trials.^33^ However, despite these multiple strong features, there are some limitations to the current set of functional capacity measures. The relationship of these measures to cognitive change may be indirect.^29^ Most of these measures are delivered in a paper and pencil format, which is not practical for remote delivery or for simultaneous assessment of multiple cases. Further, these measures are comprised of several functional tasks that are not required consistently across different cultures and do not have alternate forms.^37^ Because many of these measures were developed to assess the severity of disability, patients who are high functioning may perform near perfectly at baseline and thus cannot demonstrate improvement.

#### Interview-Based Measures of Cognition.

Many clinicians who might wish to evaluate the effect of treatment on cognitive impairment in their patients with schizophrenia do not have the required expertise or resources to conduct the meaningful performance-based assessments discussed above. Furthermore, the interpretation of the clinical relevance of changes in performance-based measures is not immediately accessible to non-experts, including clinicians, consumers, and family members, and may require different approaches or supplemental assessments with greater face validity. Clinicians may prefer an assessment that they can utilize to assess cognitive change in their patients in situations where performance-based cognitive tests are not practically available. Interview-based assessments have the potential to meet these requirements.

The strengths of interview-based assessments of cognition such as the Measure of Insight into Cognition,^39^ the Schizophrenia Cognition Rating Scale (SCoRS),^40^ or the Cognitive Assessment Interview (CAI)^41^ are brief administration time, requiring about 15min per interview,^33,41,42^ relation to real-world functioning,^25,42,46^ good test-retest reliability, and correlations with at least some performance-based measures of cognition.^12,25,33,40,42,46^ However, several challenges remain. Many studies have found no relation of interview-based measures to objective cognitive and functional measures.^39^ Due to the difficulties that patients with schizophrenia have reporting accurate information regarding cognition and everyday functioning,^43,46^ the validity of the interview-based measures and their correlations with performance-based measures of cognition may depend upon the availability of an informant. Because some patients with schizophrenia may not have contacts who know them well and are available to be interviewed,^44,47^ requirements for informant information may reduce the practicality of interview-based methods of assessment. Finally, while training demands are less than comprehensive performance-based cognitive assessment, some training is required.

#### Interview-Based Assessment of Real-World Functioning.

The domains of community functioning that are normally assessed are social functioning, vocational or non-vocational productive functioning, and residential independence and self-care. These aspects of functioning are assessed by self-report, through the involvement of relatives, friends and caregivers who provide information as informants, and clinicians. This information can also be obtained through reliable records and accurate archival materials. Important milestones for real-world functioning include obtaining, maintaining, or advancing employment; achieving residential independence; and marriage. Sub-threshold activities include taking steps toward these milestones such as seeking a job, receiving training that may enhance the likelihood of employment, or attending group social activities.

There are several different functional status rating scales that are used to examine these aspects of everyday functioning. A comparative head to head study^19^ suggested that the Specific Levels of Functioning^48^ was the best scale for identification of aspects of everyday functioning when the reference point was performance-based measures of cognition and everyday functional skills. Follow-up analyses of the database^44^ revealed that most of the 6 functional scales examined yielded ratings that were acceptably correlated with performance-based measures. An important caveat, however, is that ratings that were solely dependent on the self-report of patients or the reports of friends or relatives with a non-caregiver relationship to the patient yielded ratings with minimal validity. These data, consistent with interview-based measures aimed at cognition, suggest that the assessment of everyday outcomes requires an approach that involves more detail than just asking questions to the patient. At the same time, clinicians, who have frequent contact with a patient, including case managers and psychiatrists, generate everyday functioning ratings that are quite convergent with other assessments based on performance and ratings of cognitive functioning. One of the main drawbacks of the use of real-world functioning assessments to measure treatment response is that changes in these outcomes, unlike changes in cognition and functional capacity, are likely to take far longer than a short treatment trial. This is the main reason that the FDA does not require changes in functional outcomes for approval of cognitive-enhancing medications in schizophrenia.^45^

## Results

### Discussion and Voting of the Panelists on Relevant Issues

In order to address which of these methods are best used in the clinical assessment of cognitive treatment response, several issues were debated. This was followed by a vote by the 23 panelists. The viewpoints and voting of the panelists will be summarized here and described in detail in an online supplementary appendix. A 7-point Likert scale was used on all questions discussed. The anchors were 1 = completely agree and 7 = completely disagree. As data are presented, agreement on a particular point or question included responses 1–3; while disagreement included responses 5–7. Response 4 was considered neutral. Audience participant responses are noted only when substantially different from the panelists. A summary of voting at the meeting on outcome measure preferences is displayed in table 2.

**Table 2.** Panelist (*N* = 23) Voting Summary on Cognition Assessment Questions

Issue or Question	Mean Vote^a^	Level of Agreement (Vote 1–3)	Recommendation
Is efficacy defined as improvement in cognition?	2.7	83%	No consensus
Is efficacy defined as improvement in functioning?	2.5	67%	
The impracticality of formal cognition testing outweighs their validity for monitoring in clinical practice	4.4	39%	No consensus
Patient interviews are adequate to assess treatment response	5.5	30%	High-contact clinicians can assess treatment response; patient interviews alone are not adequate
The perspective of a reliable informant is vital to the assessment of treatment response	3.5	48%	
High-contact clinicians are able to reliably assess functional outcomes	2.7	83%	
Very brief (<5min) assessments of cognition represents the maximum that a community psychiatrist can devote	Rank ordered (see Figure 1)		No consensus
Brief assessments (<10min) of cognition will adequately assess cognition in the clinical setting			
Self-administered tests of cognition represent the optimal balance of time, effort, training, and feedback			
Performance-based measures of functional capacity, including computerized simulations, provide more information than measures of cognition and take about the same amount of time and effort			
An interview-based assessment of everyday functioning or cognitive functioning provides an assessment of the ultimate goal of treatment and a confirmation of the clinical relevance of improvement			
Breadth (more domains) vs depth (more trials per domain) is the most important aspect of cognitive performance testing for evaluation treatment response in a clinical setting	47.5% support breadth		No consensus
	30.4% support depth		

^a^Voting on a 1–7 Likert scale, with 1 = full agreement and 7 = no agreement.

#### Cognition vs Functioning.

This discussion addressed the issue of whether improvements in cognition or functioning defined a response to treatment. (Functioning refers to a patient’s ability to execute activities in the community.)

The arguments made in favor of cognition noted that the treatment would putatively be approved for improvements in cognition and that the clinical evaluation should be consistent with the labeling language. Determinations about whether the drug is effective should be made in a way that is consistent with the indication, which is cognitive impairment. Because many sources influence functioning, an evaluation of the drug should be circumscribed to cognitive effects. Because cognitive benefit may not necessarily lead to functional changes, it will be important for the prescriber to know if the drug is having an effect on cognition and to be able to make medication decisions due to lack of efficacy in an individual patient.

The arguments made in favor of a focus on functioning are that because the ultimate goal of treatment is to improve patients’ lives, clinical evaluation must emphasize improvements made in their ability to perform in the community. Baseline assessment of a patient’s goals and reasons for wanting to receive treatment can facilitate an ongoing consideration of whether a patient is making progress toward meeting the goals of treatment.

##### Consensus

There was a lack of consensus by the experts on the question of whether cognition and functioning are of primary importance in the evaluation of efficacy for a cognitive-enhancing treatment. Both were seen as important.

#### Formal Cognitive Assessment: Strengths, Weaknesses, and Alternatives.

This debate centered on whether formal assessments of treatment response are warranted or necessary or whether informal assessments of treatment response are sufficient. The arguments in favor of formal performance-based assessment of cognitive change were that these measures are objective, reliable, and less susceptible to biases such as placebo and halo effects than subjective or self-report measures. Standardized criteria for cognitive improvement and sensitivity to change could be established in order to facilitate the use of these measures. Further, while clinicians may not currently utilize them, if they become convinced that these tools are an important component of their clinical practice, they may learn how to implement and interpret them and thus change the way they practice. In-office testing is feasible and is often completed in many areas of medicine where laboratory procedures such as blood tests, physiological measures, and imaging are a part of the standard understanding of a patient’s condition and response to treatment.

Web-based tools such as “test my brain” may be useful. These web-based methods establish large databases from volunteer users that can serve as population norms. Recent work comparing web-based databases with laboratory data suggests that there are surprisingly few differences between them.^49^ Further, patients appreciate receiving this source of feedback, and it can be highly cost effective, with very little burden on clinicians. If web-based assessment proves to be valid and can be included as part of the assessment conducted in the late stages of drug development, it would reassure payers. If this testing is reimbursed, it is far more likely to be used in clinical practice. Among the drawbacks of this recently developed approach, however, are that the reliability and validity of these types of assessments have not been established in schizophrenia patient populations, and the role of clinicians and the potential burden on them may be similar to that of traditional cognitive performance testing.

The arguments against formal performance-based assessment of cognitive change in clinical practice were that (1) required cognitive testing is inconsistent with current standards of care in psychiatry; (2) it would be difficult or impossible to provide prescribers with meaningful guidelines for how to interpret changes in cognitive tests; and (3) time would be better spent evaluating client perspectives on cognitive functioning and measuring functional outcomes.

Formal evaluation with performance-based tests is not required nor routinely conducted in the evaluation of response to medications targeting other psychiatric domains such as depression, anxiety, psychotic symptoms, and negative symptoms. Even for diseases where cognitive impairment is the accepted treatment target such as Alzheimer’s disease or Attention Deficit Hyperactivity Disorder, formal performance-based evaluation is rarely completed and would not have an impact on treatment decisions. Mandated cognitive testing would likely increase the cost of treating cognitive disability in persons with psychiatric disorders. Such testing would impose an additional burden on prescribers, which could lead to fewer clients receiving these medications than would otherwise benefit, as has long been the case of clozapine in the United States. Further, as current psychological assessment guidelines require the assessments to be performed by qualified psychologists, the many clinical sites that lack a psychologist could be forced into a situation where the requirements force noncompliance with professional standards.

##### Consensus

Regarding the question of whether formal assessments of treatment response are warranted or necessary or whether informal assessments of treatment response are sufficient, consensus was not reached among the panelists.

#### Interview-Based Measures: Strengths, Weaknesses, and Alternatives.

This section was divided into 3 perspectives. The first perspective was that patient interviews are sufficient to assess response in situations where cognition must be subjectively assessed. In order to achieve this goal, however, clinicians must have historical and ongoing knowledge of the patient and understand that schizophrenia is a multidimensional disorder. The extent of experience a clinician has with similar patients will enable him or her to compare among patients. Moving forward, systems of care must provide ongoing education for clinicians regarding multidimensional assessment. Finally, training of mental health professionals and prescribers must be revamped to emphasize multidimensional assessment.

The second perspective was that because patient reports about cognition are often unreliable and uncorrelated with cognitive performance testing, the perspective of a reliable informant such as a friend or family member is vital to the assessment of cognitive treatment response. In contrast to patient reports, correlations between cognitive performance and informant reports tend to be between *r* = .3 and .4, which are medium effect sizes and suggest that informants can report on cognitive impairment in the patients they know. Most importantly, analyses of clinical trials data testing the efficacy of a cognitive-enhancing compound suggest that the addition of informants in the use of interview-based cognitive assessments may enhance the sensitivity of the measure to treatment response.^50,51^ This area remains in a nascent stage, and new data continue to emerge on the capacity of training and assessment conditions to affect the sensitivity of patient- vs informant-interviews and the relative value of various informants such as parents, siblings, friends, and caseworkers.

The third perspective was that clinicians with a high frequency of contact with the patient such as therapists or case managers are able to assess functional outcomes reliably. Many patients have difficulty reliably reporting on even simple concrete behavior such as whether they are currently employed and mood symptoms account for greater variability in scores than cognitive impairment. However, the scores that high-contact clinicians derive from the same functional outcomes in their patients are strongly correlated with patients’ severity of cognitive impairment. One of the clear challenges with this approach is that some systems of care do not enable any clinicians to have a high frequency of contact.

##### Consensus

There was strong consensus that clinicians can assess response if they have frequent contact with the patient and that patient interviews alone are not sufficient. The role of informants is important but depends upon the frequency of contact with the patient and the nature of the relationship with the patient.

### Further Considerations of Assessment Methods and Time Constraints

Several presentations and discussion focused on the strengths and weaknesses of specific methods of assessment based upon the resources and time that are available to clinicians. A summary of these discussions is presented in the supplementary appendix.

#### Review of Prioritization Voting.

Based on the discussion of assessment methods throughout the meeting, panelists were asked to rank order each of the following methods for their value in assessing cognitive treatment response in a clinical setting: comprehensive cognitive performance assessment (1–2h of testing); brief cognitive performance assessments (15–30min); briefer cognitive performance assessments (<10min); very brief (<5min) cognitive performance; self-administered tests of cognition; performance-based measures of functional capacity; and interview-based assessment of everyday functioning or cognitive functioning. Scoring methods are described in the supplementary appendix.

**Fig. 1.**
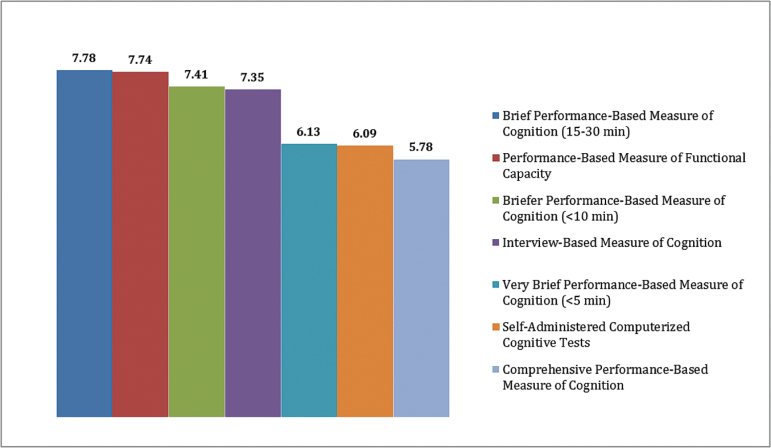
Weighted evaluation of assessment methods.

The results (see figure 1) were noteworthy for having 4 methods with similar scores at the top of the rankings and 3 with similar scores at the bottom of the rankings. Very brief, self-administered, and comprehensive cognitive assessments were not supported by the group in general, although there was considerable spread in scores and some panelists did rank one or more of these methods. Overall, however, the other 4 methods, brief (15–30min) cognitive assessments, performance-based measures of functional capacity, briefer (5–10min) cognitive assessments, and interview-based measures of cognition and functioning, were more highly ranked.

##### Consensus

Consensus on prioritization of methods was not reached. As reflected by the discussion, there was ongoing disagreement among the panelists regarding the best methods for assessing cognitive treatment response. Community and clinical psychiatrists emphasized the need for brief assessments, while academicians and psychologists emphasized the poor psychometric characteristics of these methods, and favored more rigorous methods requiring greater patient and staff time.

### Patient Selection

As with any treatment for a new indication, treatment guidelines and answers to key treatment questions should arise from leaders in the field in a consensus document. This is certainly applicable in the case of CIAS. Despite high anticipation for medications to address this huge unmet need, many clinicians may have questions about ideal patient types to be treated. For example, is everyone with schizophrenia a candidate for treatment? If not, how are treatment initiation decisions made? The discussions focused on the questions of whether patient selection should be based on:

- age and duration of illness
- baseline level of cognition
- baseline level of everyday functioning
- severity of positive symptoms

These are topics for which no studies have been done and few data exist. There are several key stakeholders with interests in answers to these questions, including payers. In the absence of product-specific data to guide answers to these questions, the panel debated these points in general terms. A summary of panelist voting is shown in table 3.

**Table 3.** Panelist Voting (*N* = 23) Summary on Cognition Treatment-Related Questions

Issue or Question	Mean Vote^a^	Level of Agreement (Vote 1–3)	Recommendation
Treatment should be initiated regardless of age or duration of illness	1.9	91%	Age or duration of illness should not be a consideration in patient selection
*If* age and chronicity are considered, younger, less chronic patients should be treated in favor of older, more chronic patients	2.2	78%	N/A
Treatment should be initiated independent of a patient’s baseline level of cognitive impairment	2.5	78%	Level of severity of cognitive impairment should not be a consideration in patient selection
*If* level of severity is important in patient selection, less impaired patients should be selected in favor of more severely impaired patients	3.7	31%	N/A
Treatment of cognitive impairment in clinical practice should be initiated independent of a patient’s level of everyday functioning	3.3	61%	Treatment should be initiated in patients independent of their opportunity to improve functionally
If baseline level of functioning is considered, treatment should focus on patients with lower levels of everyday functioning	4.6	13%	N/A
Medication treatment of cognitive impairment should be restricted to patients whose positive symptoms are stable and low/moderate	5.2	22%	Treatment can be initiated with or without the presence of low-moderate or relatively unstable positive symptoms
Nonpharmacological treatments will provide substantive benefits to drug treatments	2.3	87%	Cognitive remediation is likely to enhance drug treatment benefit, but should not be required for drug treatment to be initiated
Nonpharmacological treatments are an essential component of cognitive enhancement	3.4	56%	
Drugs labeled for adjunctive use with cognitive remediation would discourage use of these medications	2.2	82%	

^a^Voting on a 1–7 Likert scale, with 1 = full agreement and 7 = no agreement.

#### Patient Selection With Respect to Age/Duration of Illness.

This debate centered on whether age and/or duration of illness should be a determinant in selecting patients for initiation of treatment. For sake of discussion, a “younger” patient is someone with the illness for 10 years duration or less. Two contrasting viewpoints were presented: (1) younger patients or those earlier in the course of their illness should be considered for treatment vs those with longstanding illness and (2) treatment should be initiated regardless of age or duration of illness.

Arguments for suggesting that younger patients may be more amenable to treatment were (1) altered neurobiology in the aging brain affords reduced likelihood or opportunity for neuroplasticity and thus recovery of function in older people^52^; (2) younger people are more proximal to job, school, or other type of community activity, so less retraining would be involved; and (3) younger people are consequently more likely to demonstrate improvements in real-world activities like returning to work. On the other hand, as long as the medication in question does not demonstrate preferential effects in younger vs older people, a person’s age should not be a factor in patient selection. A key supporting argument is the lack of data to infer that younger patients will be more functionally responsive, which has not been shown in cognitive remediation studies. A second point is that depriving patients with longstanding disease of a potentially effective medication may be viewed as unethical.

##### Consensus

There was clear consensus that age and duration of illness should not be a consideration in patient selection for procognitive treatments. However, if resources are limited, the panelists viewed younger and less chronic patients as a priority.

#### Patient Selection With Respect to Level of Cognitive Impairment.

The discussion on patient selection with respect to baseline level of impairment was focused on whether there should be predilection for selecting patients with a higher level of cognition or functioning. The assumption is the treatment effect of the intervention does not have a preferential effect based on level of cognition. The principal arguments in support of such a position are that the less impaired patients (1) simply need a little boost to get them “over the hump” to better community functioning and (2) can more easily demonstrate functional improvements than persons with greater impairment. Further to the second point, the potential functional improvement in a patient who requires a substantial amount of support, unless the effect of the intervention is truly large, is unlikely to result in a change in dependency status. There were also arguments in support of preferentially selecting for treatment patients with greater levels of impairment: (1) ethics—depriving an effective medication from a severely ill segment of the population is unethical and (2) cognitive and functional improvements in persons with low levels of cognition may actually be easier to perceive than in those with higher cognitive ability. An analogy to this is the use of clozapine in treatment-resistant patients where improvement is easy to detect. Furthermore, functional milestones are applicable to all patients, regardless of their cognitive ability. Milestones of a person with low functional ability are every bit as meaningful as those in patients with high functional ability.

##### Consensus

Which patients receive treatment should not depend upon their baseline level of cognitive impairment.

#### Patient Selection Based on Opportunity to Functionally Improve.

The panel discussed the selection of patients for treatment based on their opportunity to improve functionally, meaning they are in a work, living, training/education, or social situation that afforded opportunities to implement skills to improve their functioning and/or are receiving psychosocial rehabilitation targeting these commonly espoused recovery goals. The contrasting perspectives were (1) patient selection should favor those who have an opportunity to improve functionally because functional improvement is the primary goal of treatment and psychosocial rehabilitation programs can be a potent moderator of cognitive benefits from cognitive enhancement and (2) most patients can be treated regardless of their opportunity to improve functionally because noticeable improvement in one aspect of cognition defines efficacy. The principal argument in support of the first perspective was that opportunities or situations to improve functionally, such as a job, school, or even management of daily living skills, enable patients to exercise their cognition. Those patients who have no opportunities to make functional changes or are not engaged in psychosocial rehabilitation programs will not benefit sufficiently to make treatment valuable,^9,53^ and lack of functional opportunities may limit the extent to which cognitive change improves everyday functioning.^29,54^

On the other hand, it can be argued that most patients should be treated regardless of opportunity to improve functionally because of (1) the lack of precision in identifying potential responders; (2) a wide variation among patients may exist in terms of what defines a functional response, with many patients facing functional challenges that are immutable; and (3) withholding an approved treatment from someone who lacks an opportunity to functionally improve may be viewed as unethical. All patients who have a need to improve functionally warrant a therapeutic trial with a treatment.

##### Consensus

Treatment should be initiated in patients independent of their opportunity to improve functionally.

#### Patient Selection Based on Stability and Extent of Positive Symptoms.

Clinical studies evaluating the procognitive effects of potential medications in the treatment of cognitive impairment identify only subjects who meet certain stability criteria for enrollment. Predominant among these selection criteria are that positive symptoms are no worse than mild-moderate and that they are relatively stable. Additional criteria are stable doses of background antipsychotic medications, minimal extrapyramidal symptoms, and no symptoms of depression. The primary purpose of these criteria is to ensure that changes observed in cognition are attributed to the intervention (study drug) and not changes in other facets of their disease. With these stability criteria, the applicability of efficacy data to the entire population of patients, particularly those whose symptoms are less stable, could be questioned. Therefore, the panelists debated whether procognitive treatments should be limited to relatively stable patients with low levels of positive symptoms. Further debate was held on the fate of treatments during periods of positive symptom acute exacerbation. The purpose of this discussion was to formulate recommendations to the field on prescribing medications to patients whose symptom levels were outside the scope of those persons with schizophrenia who had participated in cognitive enhancement clinical trials.

Arguments made in support of limiting treatments in patients whose symptoms are stable and low were mainly limited to initiation of treatment, not maintenance. If there were no data to support the benefits of treatments in patients with fluctuating symptoms, the cost and safety risks, depending in the profile of the medication in question, may not justify the prescription. There was no support among the panelists for discontinuing treatment if a patient’s symptoms worsened or became unstable after starting treatment. The points made in support of treating a broad group of patients irrespective of the degree of positive symptoms were (1) little correlation between positive symptoms and cognition; (2) the likely targets for cognitive-enhancing drugs are independent of psychosis; (3) effective cognition treatments will likely require continuous dosing, rather than stopping and starting with symptom fluctuations; and (4) successful treatments for cognitive impairment may enable some patients to participate in programs that otherwise would not be possible.

##### Consensus

Treatment can be initiated in a patient population that is likely to respond to treatment, with or without the presence of low-moderate or relatively unstable positive symptoms. Furthermore, procognitive medications need not be discontinued during periods of acute exacerbation of psychosis.

#### Use of Procognitive Medications With Nonpharmacological Treatments.

Because there are no approved pharmacological methods of treating CIAS, no data exist on the combination of cognitive remediation or behavioral therapy with pharmacological treatments. Of course, final decisions about implementing cognitive remediation strategies during clinical trials and during clinical practice will be determined by several important factors, including the mechanism of action of the treatment and the perceived need for experience-based learning to be used alongside novel pharmacology. The discussion and voting at the meeting were completed not to preempt these important issues but to understand the current thinking on these topics from experts in this area of work as well as from industry leaders who may be considering the inclusion of cognitive remediation treatments in their future clinical trials. Therefore, the panel addressed the necessity and uncertainty of combining nonpharmacological and pharmacological treatments on patient outcomes. Issues related to the strength of the dependence of pharmacologic treatment on the presence of behavioral treatment were discussed at 3 different levels: (1) will nonpharmacological treatments provide substantive benefits to drug treatments; (2) will nonpharmacological treatments be an essential component to drug treatment; and (3) if nonpharmacological treatments are a necessary adjunct to drug treatment, what will be the impact on drug prescribing?

Cognitive remediation has a high potential to increase the efficiency of drug treatments.^55,56^ Meta-analyses indicate the general treatment effect size associated with cognitive remediation therapy is approximately 0.36–0.45.^9,53^ Pharmacologic treatment may have the potential to address lower level cognitive functions in a more efficient manner than cognitive remediation. The combination would enable cognitive remediation to focus on higher level cognitive processes, such as problem solving, that may not be as amenable to pharmacotherapy. Cognitive remediation can then promote generalization of cognitive benefit by teaching how to use the pharmacologically enhanced skills to perform the multidimensional cognitive tasks.

It can be argued that nonpharmacological treatments are an essential component of cognitive enhancement. Some believe, although supportive data do not exist, that administering a pharmacologic treatment without retraining or education is futile. The rationale for this hypothesis is that medications are likely to improve a patient’s cognitive abilities but daily activities may not include tasks that foster these abilities. A close analogue is d-cycloserine in the treatment of anxiety disorders, where data suggest that training is necessary for behavioral gains to be realized.^57^ The counter argument to this perspective is that because the improvements resulting from behavioral cognitive remediation and pharmacologic treatment may be overlapping, utilizing both treatments at the same time is redundant and unnecessary.

The impact on prescribing procognitive drugs was discussed under the assumption that studies will show procognitive medications are effective only in combination with cognitive remediation, and this would be reﬂected in the label. Much of this impact was dependent on (1) practice setting/prescriber and (2) patient access to cognitive remediation. In an acute inpatient facility, the short stay is more conducive to cognitive assessment and psychoeducation about cognitive health than starting a course of cognitive remediation which on average runs for 32 sessions. Making cognitive remediation available on an acute unit is not likely to improve short-term outcome, reduce patient costs, or increase institutional revenue. Therefore, in acute care settings, it would be burdensome and of no benefit to require cognitive remediation in addition to prescribing a medication. However, cognitive remediation could be prescribed for initiation during outpatient treatment. In a long-term inpatient facility, treatment focuses on symptomatic stabilization and preparing the patient for community reentry. Given their longer length of stays, cognitive remediation plus medication in this situation may be cost-effective. However, in many regions of the United States and throughout the world, cognitive remediation in these facilities is largely unavailable. Therefore, access to cognitive remediation would persist as a rate-limiting constraint to prescribing. The treatment focus in day treatment and partial hospitalization programs is on symptom stabilization and optimizing community function. Initiating cognitive remediation in these facilities is certainly within the scope of practice; however, as in long-term inpatient facilities, the unavailability of cognitive remediation at this time would be a rate-limiting factor. In outpatient sites, cognitive remediation is more widely available. However, transportation, cost, and noncompliance pose huge constraints on its effectiveness.

##### Consensus

The group was clear that cognitive remediation is likely to facilitate and potentially enhance a drug treatment benefit. However, about half of the panelists believed that some form of nonpharmacological treatment is needed in combination with medication treatments; without behavioral treatment, improved cognition cannot be attained. About one-third of the panelists believed that medications were acceptable to administer without nonpharmacological therapy. Cognitive remediation should not be required for drug treatment to be initiated. The group recommended that drug companies and other developers of procognitive medications study the additive benefits of cognitive remediation and other nonpharmacological treatments in the development of procognitive medications and that data be published or included in product labeling.

## Conclusions

The panelists, who came from very diverse backgrounds in terms of career and educational focus, reached strong consensus on only a few topics. The discussions and voting were striking not only for the breadth of opinion but also for their resistance to significant change following discussion. Some of the challenge of reaching consensus was attributable to the absence of clear data to allow the creation of informed opinion, particularly with regard to the absence of an approved treatment for cognitive impairment in schizophrenia. The output from this meeting suggests that additional research on these issues is necessary, and the results reported here may provide a roadmap for future work. In addition, dissemination of this work will be essential for the various stakeholders to come to a significant alignment on the various methods for assessing cognitive change in clinical settings.

Group voting from the panelists and audience participants suggested agreement that both cognition and functioning are equally important in the evaluation of efficacy for a cognitive-enhancing treatment. This result has important implications for the development of clinical trials methodology and educational programs for clinicians. First, it will be important to know the cognitive and functional changes associated with any new treatments under evaluation, and thus the development of new treatments should include functional and cognitive outcome measures. In addition, efforts to educate clinicians on the evaluation of treatment response should include both types of outcomes.

No consensus was reached on whether the impracticality of formal assessments of cognition outweighs their validity for monitoring treatment in clinical practice. Some panelists and participants, particularly those in clinical practice with substantial time and resource concerns, favored brief assessments and a focus on interview-based techniques. Other panelists, particularly researchers and psychologists with concerns about the psychometric characteristics and validity of the outcome measures, favored longer assessments with standardized methods of evaluation. This divergence was considerable and reflects existing differences among mental health professionals in their approach to clinical evaluation. It is unreasonable to expect that these differences will be narrowed considerably. However, it will continue to be valuable for those representing these different perspectives to inform one another of relevant empirical data as they emerge.

There was strong consensus that clinicians can assess response if they have frequent contact with the patient. However, there was also strong consensus that patient interviews alone are not sufficient. There was consensus that the gaps in information provided by patients themselves can be filled by people who observe patients in their everyday lives. However, there was also clear consensus that the contribution of an informant depends upon the frequency of contact that an informant has with the patient.

Regarding the central question of which specific assessment methods are most favorable for clinical evaluation of treatment response, there was no clear consensus. Brief performance-based cognitive assessments, interview-based assessments, and performance-based measures of functional capacity were viewed as slightly more favorable than comprehensive test batteries, very brief assessment, or self-administered computerized cognitive tests.

Although the group did not reach consensus on the practicality of formal testing, a number of members expressed enthusiasm for cognitive assessments that could be administered in a clinic office with minimal training by office staff. There was agreement that it was feasible to develop cognitive tests that would be administered on a tablet or a similar device. Results could be delivered to the clinician with minimal delay. It is important to note that participants were not aware of a validated assessment that is currently available to provide this service.

There was considerable agreement among the panelists and participants on what factors should be considered in selecting patients for treatment. The group attained consensus that patient selection should not depend upon age or chronicity, baseline level of cognitive or functional impairment, or the severity of positive symptoms. However, the groups conceded that as treatments are developed, empirical data may become available that help target specific patient populations for specific treatments.

In summary, broad consensus was reached on most topics related to patient selection and treatment, and these conclusions can be usefully applied when treatments for cognitive impairment become available for people with schizophrenia. However, there was only moderate consensus on the majority of the questions addressing treatment goals and methods for monitoring cognitive deficit treatment response in clinical practice. The disagreement among participants was largely explained by the lack of solid data on these topics. The discordance observed at this conference around optimal monitoring of cognition in the clinical setting may be addressed by further development and validation of instruments to measure cognition and functioning, with the busy practicing clinician in mind as the end user. There appears to be a dearth of brief and psychometrically valid tools that can be easily used by nonpsychologist clinicians to measure cognition in their offices. How can large databases and web-based data collection facilitate this progress? Is it possible to develop tools that can be used without specialized training? Finally, it will be important to include key stakeholders—patients, families, payers—into the discussion so that these new tools can include their input about the components of cognition that are most worrisome to them.

It is important for the field to develop these tools now so that when treatments for cognitive impairment become available, the tools to monitor patient progress will be ready. The criteria for the usefulness of cognitive outcome measures in clinical trials and clinical practice have considerable overlap.^58^ As described above, these measures need to have strong test-retest reliability, validity, correlations with functional outcomes, minimal practice effects, sensitivity to diagnostic differences, and sensitivity to treatment effects. They should also be practical for testers and tolerable for patients. It is essential that any new tools for measuring cognition in clinical practice meet these criteria. They will also need to demonstrate the flexibility that is found in traditional cognitive tests. While significant promise to improve the efficiency of testing and therefore allow greater numbers of patients to be tested, these measures will need to improve upon previously high missing data rates.^21,25^ As described by Bauer *et al* ^59^ in their joint position paper of the American Academy of Clinical Neuropsychology and the National Academy of Neuropsychology, performance claims for electronic outcome measures will need to meet FDA Center for Drug Evaluation Research device regulations, with encryption for “store and forward” procedures, and proper privacy and identity verification mechanisms, preferably with biometric login systems. These devices should be configured so that they do not discriminate against patients based upon age, race, education, or socioeconomic status, which can be complicated when utilizing technologies that may be very familiar to some individuals while others have not used them. Finally, the lure of the device should not be the governing factor in outcome choice. The determination of the value of a measure should be its contribution to clinical decision making, not whether it makes the clinician feel more technologically sophisticated.

Finally, considerable progress in recent years has empirically linked cognition to community outcomes, but more work is needed to move this important area of research into the practicing clinician’s office. The authors also recommend that psychiatry training programs provide greater attention to cognitive treatments and methods for assessment so that emerging clinical psychiatrists are aware of how to address cognitive impairment in their patients with schizophrenia.

## Supplementary Material

Supplementary material is available at http://schizophreniabulletin.oxfordjournals.org.

## Funding

Funding for this manuscript was provided by the International Society for CNS Clinical Trials and Methodology.

Dr Keefe currently or in the past 3 years has received investigator-initiated research funding support from the Department of Veteran’s Affair, Feinstein Institute for Medical Research, GlaxoSmithKline, National Institute of Mental Health, Novartis, Psychogenics, Research Foundation for Mental Hygiene, Inc., and the Singapore National Medical Research Council. He currently or in the past 3 years has received honoraria, served as a consultant, or advisory board member for Abbvie, Akebia, Amgen, Asubio, AviNeuro/ChemRar, BiolineRx, Biogen Idec, Biomarin, Boehringer-Ingelheim, Eli Lilly, EnVivo/FORUM, GW Pharmaceuticals, Janssen, Lundbeck, Merck, Minerva Neurosciences, Inc., Mitsubishi, Novartis, NY State Office of Mental Health, Otsuka, Pfizer, Reviva, Roche, Sanofi/Aventis, Shire, Sunovion, Takeda, Targacept, and the University of Texas South West Medical Center. Dr Keefe receives royalties from the BACS testing battery, the MATRICS battery (BACS Symbol Coding), and the Virtual Reality Functional Capacity Assessment Tool. He is also a shareholder in NeuroCog Trials, Inc. and Sengenix. Dr Haig is a full-time employee of Abbvie. Dr Marder has received consulting fees from Abbvie, Genentech, Roche, Lundbeck, Pfizer, Otsuka, Takeda, and Boeringer Ingelheim. He has received research support from Amgen, Sunovion, and Synchroneuron. Dr Harvey has received consulting fees from Abbvie, Boehringer Ingelheim, Forest Labs, Forum Pharma, Genentech, Otsuka America, Roche Pharma, Sunovion Pharma, and Takeda Pharma during the past year. He also received contract research support from Genentech. Dr Dunayevich for the past 3 years has been a full-time employee and stockholder of Amgen. Dr Medalia in the past 3 years has received research funding support from Sunovion. Dr Medalia has also currently or in the past 3 years received honoraria or served as consultant for Dainippon Sumitomo Pharma Co., Ltd., Otsuka, and Takeda Pharmaceuticals U.S.A., Inc. Dr Davidson has received research grant support and/or travel support and/or speaker fees and/or consultancy fees from Lundbeck, Eli Lilly, Servier, Abbott, Minerva and holds stocks in CTR and BiolineRx. Dr Lombardo is a full-time employee of FORUM Pharmaceuticals. Dr Bowie reports receiving grant support from Pfizer. He has also been a consultant for Lundbeck, Otsuka, Abbvie, and Takeda. Dr Buchanan reports: Advisory Board: Abbvie, Amgen, EnVivo, Roche; Consultant: Abbvie, Amgen, Bristol Myers Squibb, EnVivo, Omeros; DSMB member: Pfizer. Dr Bugarski -Kirola is a full-time employee of Hoffmann-La Roche Ltd. Dr Carpenter in the past 2 years has been a consultant to Roche/Genetech. Dr Dago in the last 3 years has received honoraria from Lundbeck, Forest Pharmaceuticals, Otsuka, Pam Labs, and Astra Zeneca for lectures given in promotion of their psychotropic medications. Dr Durand in the past year has been a consultant and received honoraria from Teva Pharmaceuticals. Dr Gold receives royalty payments from the BACS. He also has served as a consultant for Amgen, Hoffman LaRoche, and Lundbeck. Dr Hooker has served as a consultant and is currently a Co-Investigator on an NIH SBIR grant with PositScience Corporation. Dr Loebel is an employee of Sunovion Pharmaceuticals. Dr McGurk reports receiving consulting fees from Abbvie and EnVivo Pharmaceuticals. Dr Pinkham in the past year has received consulting fees from Otsuka America Pharmaceutical, Inc. The following authors have declared that there are no conflicts of interest in relation to the subject of this study: Drs Csernansky, Frese, Goff, Kopelowic, Opler, and Stern.

## Supplementary Material

Supplementary Data
