# Supplementary material for: Report on ISCTM Consensus Meeting on Clinical Assessment of Response to Treatment of Cognitive Impairment in Schizophrenia
Source: Schizophr Bull. 2015 Sep 11;42(1):19–33. doi: 10.1093/schbul/sbv111 (PMC4681562; doi:10.1093/schbul/sbv111)
Supplement: Supplementary Data [file supp_42_1_19__index.html]

Report on ISCTM Consensus Meeting on Clinical Assessment of Response to Treatment of Cognitive Impairment in Schizophrenia — Report on ISCTM Consensus Meeting on Clinical Assessment of Response to Treatment of Cognitive Impairment in Schizophrenia — Supplementary Data 

# Report on ISCTM Consensus Meeting on Clinical Assessment of Response to Treatment of Cognitive Impairment in Schizophrenia

## Supplementary Data

Data files

- Supplementary Data - Supplementary Data
