# Supplementary material for: Report on ISCTM Consensus Meeting on Clinical Assessment of Response to Treatment of Cognitive Impairment in Schizophrenia
Source: Schizophr Bull. 2015 Sep 11;42(1):19–33. doi: 10.1093/schbul/sbv111 (PMC4681562; doi:10.1093/schbul/sbv111)
Supplement: Supplementary Data [file supp_sbv111_REVISED_ISCTM__Consensus_Meeting_Appendix_FINAL.doc]

ISCTM Consensus Meeting Appendix

*Cognition versus Functioning.*

*Review of relevant votes.*  As presented in Figures 1 and 2, the panelists were split on this question, with similar average ratings for an emphasis on functioning (2.5) and cognition (2.7); 83% agreed that cognitive change should define treatment response and 65% agreed that functional change should define treatment response. However the functioning question was somewhat bimodally distributed while the cognition question was normally distributed. A larger percentage (30%) strongly agreed that efficacy should be defined based upon response in functioning compared to 13% for cognition. However 35% were neutral or disagreed that efficacy should be defined based upon functional outcomes.

*Formal cognitive assessment: strengths, weaknesses, and alternatives.*

*Review of relevant votes.* With regard to the question of whether the impracticality of formal assessments of cognition outweighs their validity for monitoring treatment in clinical practice, as shown in FIGURE A3, the mean response of the group was neutral, with a mean score of 4.4. However, the distribution tended toward bimodality, with 39% of the panelists agreeing with that statement and 52% disagreeing, and 22% disagreeing strongly.

*Interview-based measures: strengths, weaknesses, alternatives*

*Review of relevant votes.* As demonstrated in Figures 4-6, panelists gave a score of 5.5 to the statement “patient interviews are sufficient to assess response,” with 56.5% of them giving a score of 6 or 7 (strongly disagree). Only 30.4% of the panelists agreed with the statement, none strongly. With regard to the statement of whether “informants are vital to the assessment of response,” there was very little agreement. The mean score of agreement was 3.5, with a score of 4 (neutral) receiving the highest number of votes (21.7%), and 47.8% agreeing and 30.5% disagreeing. The audience participants had a stronger agreement to the statement, with 70.4% agreeing. Regarding the statement that “a high contact clinician can determine response based upon regular examinations,” the mean score was 2.7 suggesting considerable agreement. 82.6% of the panelists agreed with the statement.

Issues with Specific Assessment Methods

Each statement represents the crux of the argument put forth.

*Very brief (< 5 min) assessment of cognition represents the maximum effort that a community psychiatrist can devote to the assessment of cognition.*  The strength of this “briefer is better” approach is that because of the unfortunate time constraints in assessing patients with schizophrenia, more time is often unavailable, and clinicians rarely use rating scales with their attendant time and training demands. While this approach has some considerable conceptual and psychometric weaknesses, if resources dictate, it is important to focus on a limited number of relevant behaviors, requiring staff members or personal informants who are open to sharing relevant information.

*Brief assessments (<10 min) of cognition will adequately assess cognition in the clinical setting.* This approach emphasizes individualized assessment based on the interests and activities of the person to be assessed, and ideally is combined with (very limited) brief objective assessment.

*Self-administered tests of cognition represent the optimal balance of time, effort, training and feedback*. This approach involves computerized assessments that are administered in the clinic office, waiting room or on-line. The benefit of this approach is that it is not susceptible to the biases and questionable relevance of patient reports and interview-based assessments, it may require less supervision, and results can be quickly compared to existing norms, interpreted, and integrated into a patient’s medical record. This approach may be particularly fruitful if tests with adaptive designs can be used. Some of the weaknesses of this approach are that they depend upon the availability of specified hardware, resources for test licenses and appropriate testing environment. Most importantly, self-assessment of cognition in patients with schizophrenia can be highly susceptible to invalid patient-test interactions without the presence of a trained tester and engaged patient. In addition, some patients, especially older patients, may be unaccustomed to tablet-based or computer-based interactions and require assistance.^59^

*Performance-based measures of functional capacity, including computerized simulations, provide more information than measures of cognition and take about the same amount of time and effort.* This approach focuses on the assessment of treatment response by determining whether a patient has had a change in his or her performance on a task that is relevant to their everyday functioning. It argues that if clinicians want to know whether a patient’s capacity to function has changed, they should assess the functions that they may need to engage in, such drawing money from a virtual-reality ATM machine or going on a virtual shopping trip. Several high quality functional simulations are in development. Some use virtual reality technology, and others use touchscreen technology to closely simulate real-world tasks.

*An interview-based assessment of everyday functioning or cognitive functioning provides an assessment of the ultimate goal of treatment and a confirmation of the clinical relevance of improvement; it therefore should be the standard form of assessment of a cognitive intervention.*  This approach emphasizes that evidence of improvement in day-to-day functioning must be obtained in order to justify the costs and risks of treatment. The levels of functioning that are assessed may range from the microscopic (skills) to the macroscopic (activities). Not only the magnitude of the improvement, but also its value to the patient and caregivers should be established. The assessment must be individualized. The skills and activities assessed must be specific to the patient. Sources of information should include the patient, caregivers, and clinic staff. The clinician, using an interview based measure, is singularly able to individualize the assessment, integrate the input from the various sources of information, and reach a global impression of improvement. The interview-based assessment also furthers the treatment alliance and yields data that informs the overall treatment plan.

An additional question was posed to compare breadth of domains versus depth of items in constructing cognitive performance test batteries: *Given a fixed amount of time for assessment, which is the most important aspect of cognitive performance testing for evaluation treatment response in a clinical setting, breadth of information (more domains) or depth of information (more trials per domain)?* Cognitive impairment in schizophrenia is broad and deficits in specific cognitive domains are differently associated with the various areas of daily functioning. Further, some pharmacologic and behavioral treatment studies have suggested that specific treatments may benefit selected cognitive domains. Therefore, cognitive assessments need to tap a breadth of functions. On the other hand, composite cognitive scores have psychometric characteristics such as increased reliability and reduced variability that make them more sensitive to change. Batteries of tests that allow for a reliable assessment of general cognitive impairment may be the best choice given the challenges of reliable change measurement discussed above. It may be that measurement strategies can be driven by what is known about the action of the drug or intervention. For example, if clinical trials suggest that attention is improved by a treatment, assessment can focus on that domain. If the drug appears to have more general effects, a broader assessment may be necessary.

*Scoring Procedures for Prioritization of Cognitive Assessment Methods.* Scores were determined using a weighted analysis system as follows: panelists voted for items as their 1st choice, 2nd choice, 3rd choice, and so on. A program examined each item, calculated the percent of people who selected this item as a 1st choice, and multiplied this percentage by a weighting factor of 10. For example, if an item on the list was the first choice of 50% of the voters, it is awarded 5 points. The system then calculates the percent of people who selected this item as a 2nd choice, and multiplies this percentage by a weighting factor of 9. This continues until the maximum number of choices is reached. The weighting factor declines each time by 1 point. Points for this item are then totaled and are presented in FIGURE A7.

*Patient Selection with Respect to Age/Duration of Illness*

*Review of relevant votes.* In one of the most widely supported questions addressed by the panel, a vast majority of panelists (91.3%) voted “1”, “2”, or “3” in agreement that treatment of cognitive impairment in clinical practice should be initiated independent of a patient’s age and chronicity of illness (FIGURE A7) Only 8.8% of respondents disagreed. The mean score of panelists was 1.9, which was the lowest mean score (greatest support) of all questions addressed. The audience participants were nearly equally supportive, with 75.6% in agreement.

The follow-up question, “If age and chronicity are considered, should treatment of cognitive impairment in clinical practice focus on younger, less chronic patients or older, more chronic patients?” drew a large percentage of votes (78.3%) for younger patients from the panelists. Responses of the audience participants was similar, with 60.0% of respondents also suggesting treatment initiation in younger patients vs. older patients if there had to be a choice, with a sizable portion (37.8%) of “4” (neutral) score.

*Patient Selection with Respect to Level of Cognitive Impairment*

*Review of relevant votes.* A substantial majority of panelists agreed that procognitive treatment should be initiated regardless of a patient’s level of impairment (FIGURE A8). 78.2% indicated agreement with the statement, with 8.7% giving a score of 4, indicating neutral. The audience was slightly higher at nearly 82.3% in support of the statement. When asked which patient group (less impaired or more impaired) should be preferentially selected if baseline level of cognition is a determinant in patient selection, there was an even split within the expert panelists. Slightly more than 30% supported targeting treatment for less impaired (scores of 1-3 for less impaired), compared with 26% suggesting targeting more impaired patients. Nearly 44% of panelists scored a “4”, indicating lack of preference for level of impairment. Audience participants were more in favor (35.6%) of less impaired patient selection vs. more impaired (11.0%), with more than 50% scoring a neutral “4.”

*Patient Selection Based on Opportunity to Functionally Improve*

*Review of relevant votes:* More than 60% of the panelists thought that procognitive treatments could be given to patients regardless of their opportunity to improve functionally, while nearly 35% were opposed and 4.4% were neutral (FIGURE A9). The audience participants were in stronger support of providing treatment regardless of opportunities to improve with 73.3% of respondents in support and 11.1% neutral.

*Patient Selection Based on Stability and Extent of Positive Symptoms*

*Review of relevant votes.* The overwhelming majority of panelists (nearly 74%) supported broad treatment of patients regardless of the level of positive symptoms (FIGURE A10). Only 21.8% of panelists believed treatments should be restricted to stable and low level symptom patients.

*Use of Procognitive Medications with Non-pharmacological Treatments*

*Review of relevant votes:* A large majority of panelists (86.9%) and audience participants (77.3%) agreed that cognitive remediation is likely to provide substantial benefits in combination with procognitive medications (FIGURE A11). Nearly 56% of the panelists believed that some form of non-pharmacological treatment is needed in combination with medication treatments; without behavioral treatment, improved cognition cannot be attained (FIGURE A12). On the other hand, 31.2% of the panelists believed that medications were acceptable to administer without non-pharmacological therapy. The vast majority (87.0%) of panelists responded that the requirement of cognitive remediation would discourage the use of procognitive medications (FIGURE A13). A similar proportion of audience participants (82.3%) also had the same view.

**FIGURE A1**


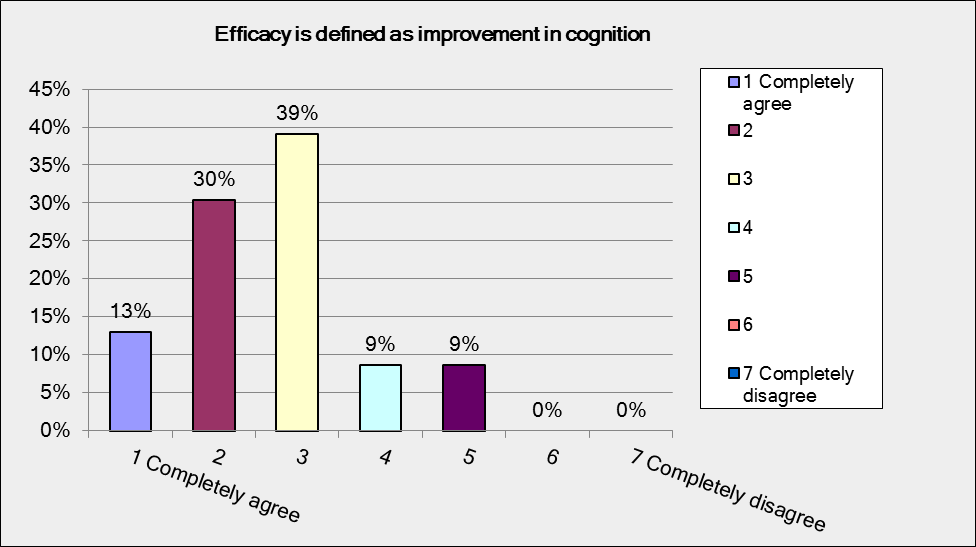


**FIGURE A2**


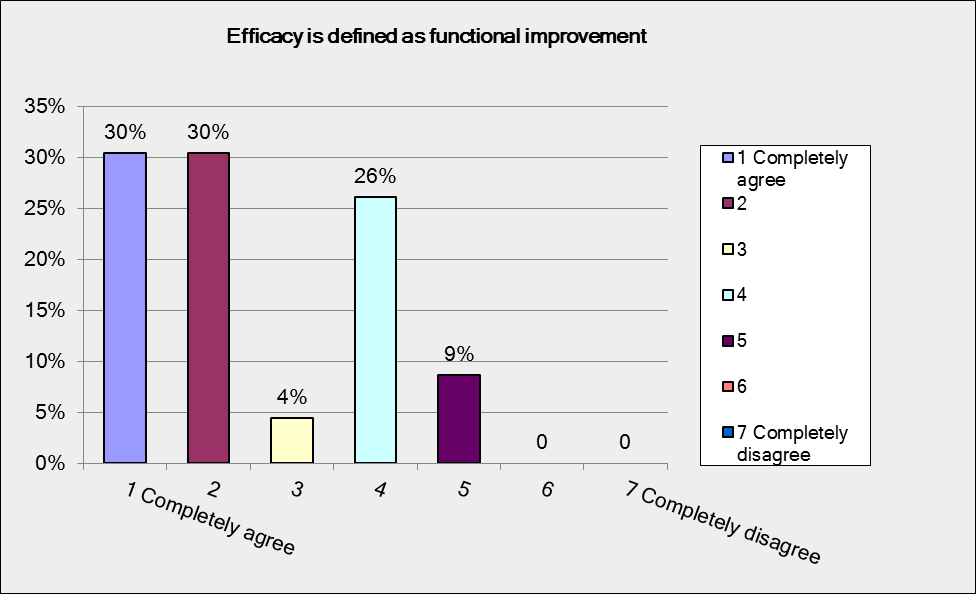


**FIGURE A3**


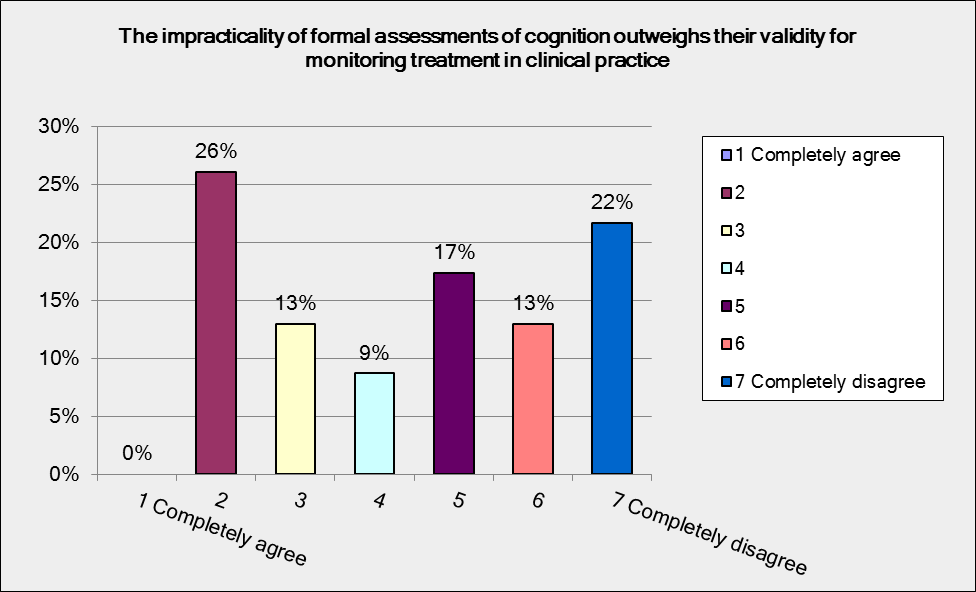


**FIGURE A4**


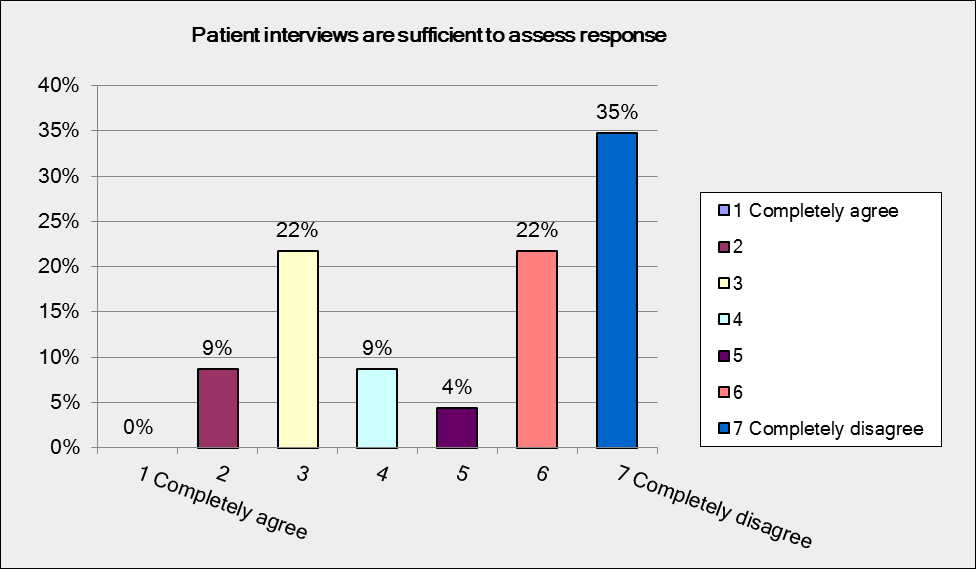


**FIGURE A5**


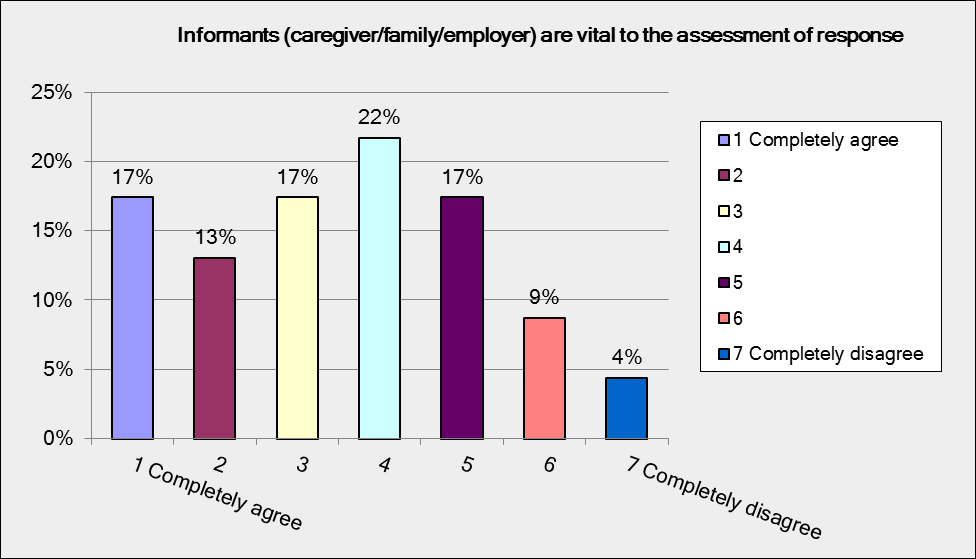


**FIGURE A6**


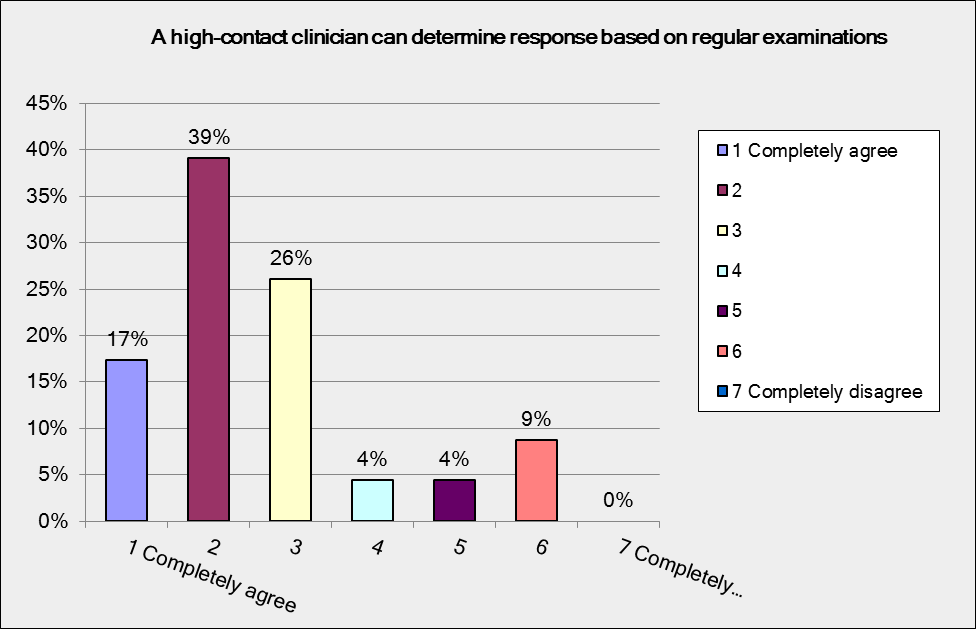


**FIGURE A7**


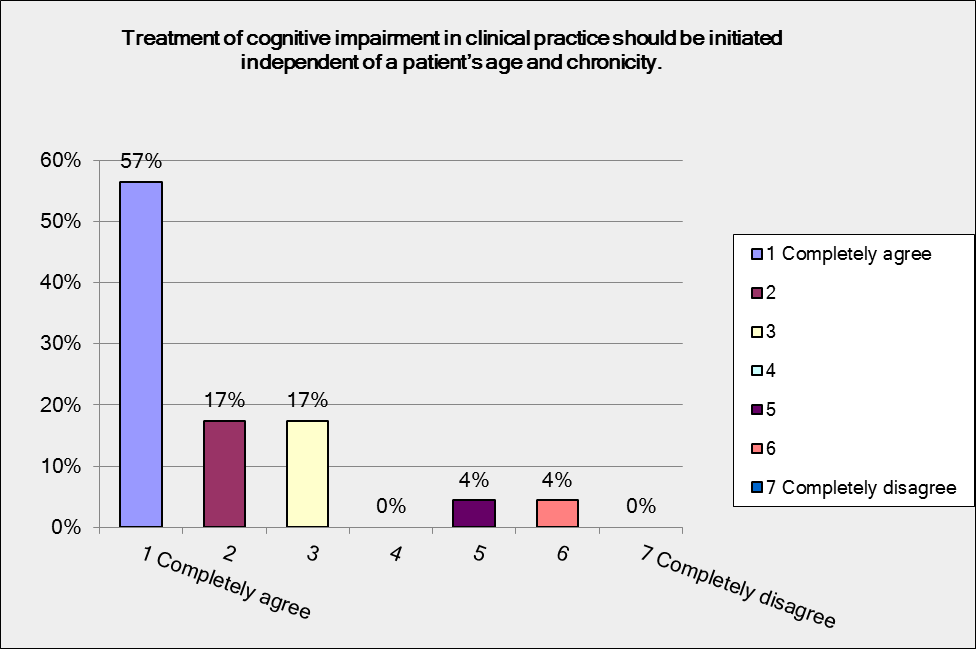


**FIGURE A8**


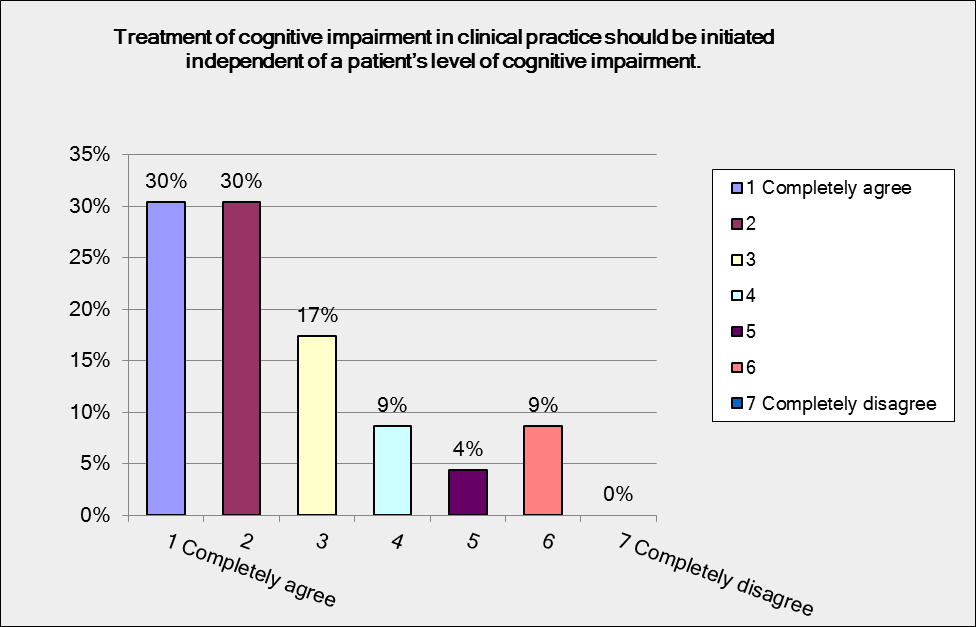


**FIGURE A9**


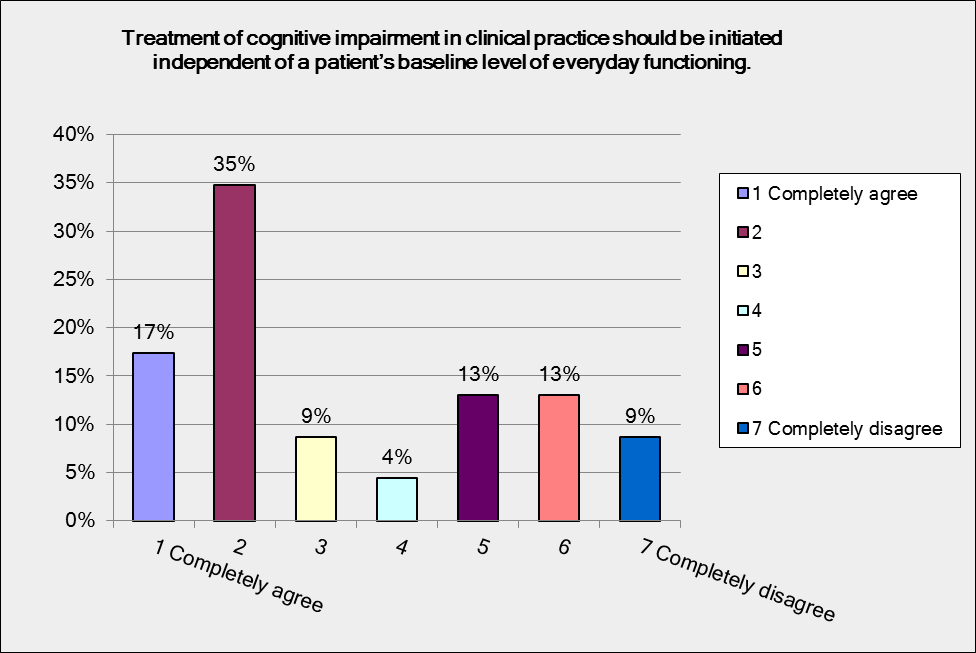


**FIGURE A10**


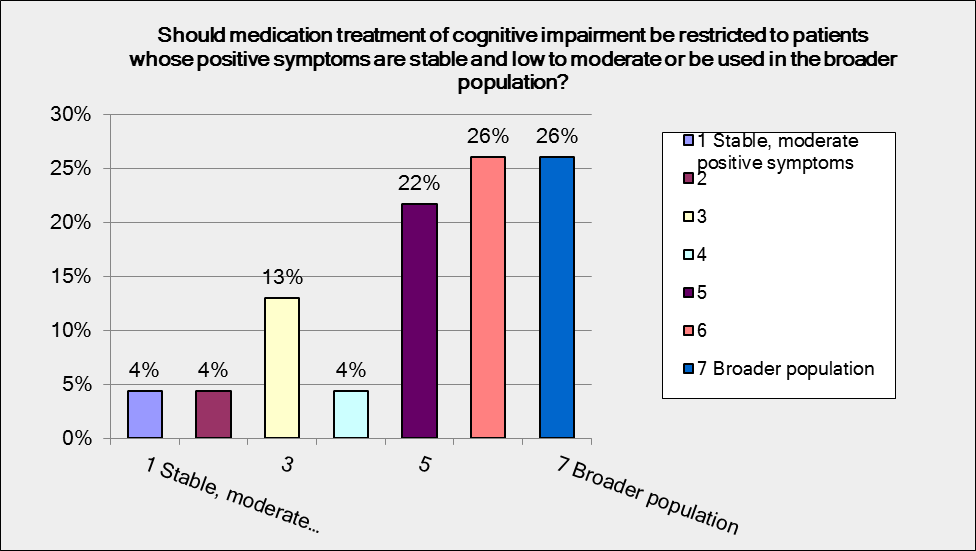


**FIGURE A11**


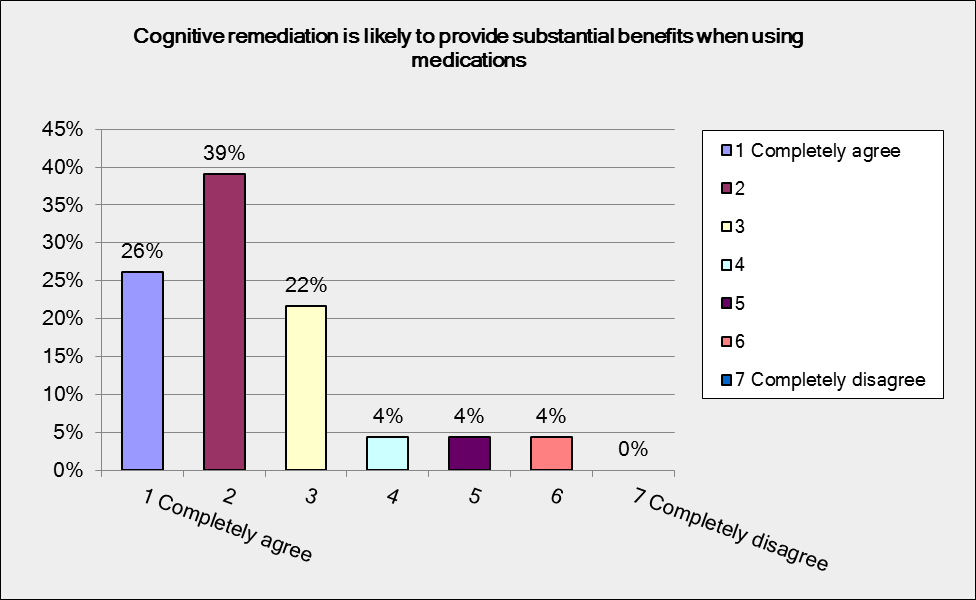


**FIGURE A12**


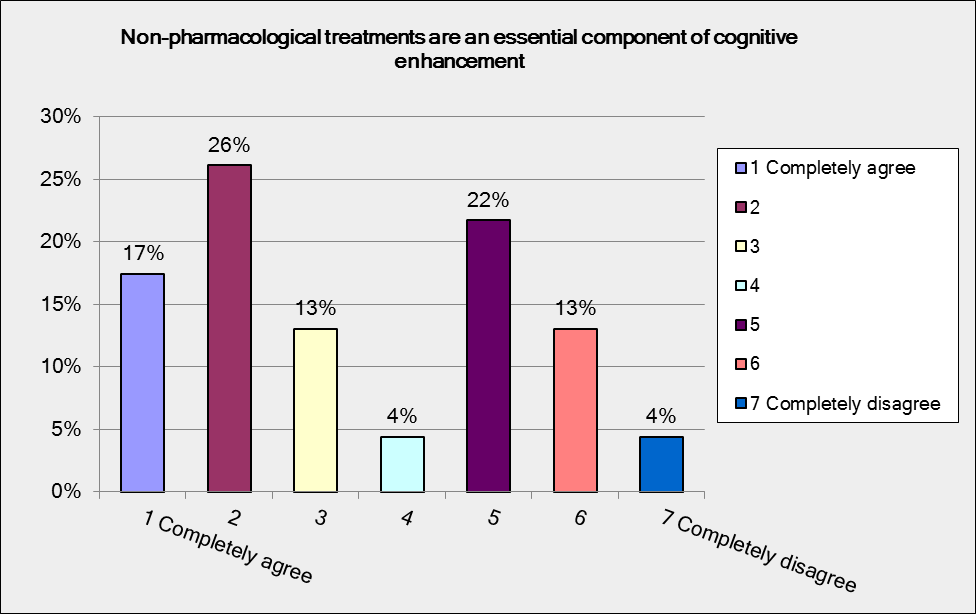


**FIGURE A13**


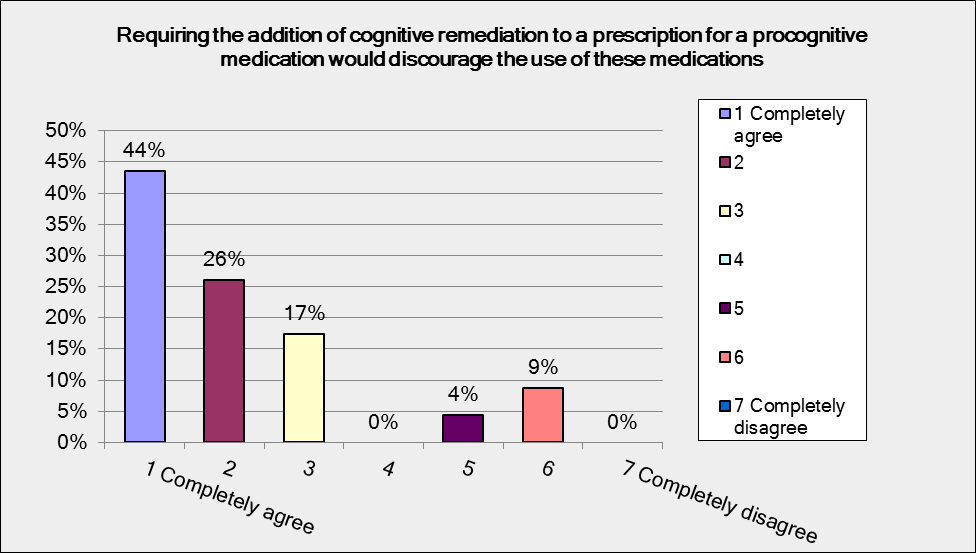


Questions for which pre-meeting questionnaire reflected existing consensus.

4. Rate these characteristics of cognitive assessments in terms of how important they are in considering how to assess response to treatment (1 = very important; 7 = not at all important):

| **a. Amount of time and effort required from the patient** | | | | | | |
| --- | --- | --- | --- | --- | --- | --- |
| **Answer Options** | | **Response Percent** | **Response Count** | | | |
| 1 very important | | 23.5% | 8 | | | |
| 2 | | 29.4% | 10 | | | |
| 3 | | 32.4% | 11 | | | |
| 4 | | 5.9% | 2 | | | |
| 5 | | 5.9% | 2 | | | |
| 6 | | 0.0% | 0 | | | |
| 7 not at all important | | 2.9% | 1 | | | |
| ***answered question*** | | | **34** | | | |
| ***skipped question*** | | | **0** | | | |
|  |  |  |  | | | |
| **b. Amount of time and effort required for the physician** | | | | | | |
| **Answer Options** | | **Response Percent** | **Response Count** | | | |
| 1 very important | | 64.7% | 22 | | | |
| 2 | | 32.4% | 11 | | | |
| 3 | | 0.0% | 0 | | | |
| 4 | | 2.9% | 1 | | | |
| 5 | | 0.0% | 0 | | | |
| 6 | | 0.0% | 0 | | | |
| 7 not at all important | | 0.0% | 0 | | | |
| ***answered question*** | | | **34** | | | |
| ***skipped question*** | | | **0** | | | |
|  |  |  |  | | | |
| **c. Amount of time and effort required by non-physician staff (e.g., nurse, psychologist)** | | | | | | |
| **Answer Options** | | **Response Percent** | **Response Count** | | | |
| 1 very important | | 20.6% | 7 | | | |
| 2 | | 38.2% | 13 | | | |
| 3 | | 38.2% | 13 | | | |
| 4 | | 2.9% | 1 | | | |
| 5 | | 0.0% | 0 | | | |
| 6 | | 0.0% | 0 | | | |
| 7 not at all important | | 0.0% | 0 | | | |
| ***answered question*** | | | **34** | | | |
| ***skipped question*** | | | **0** | | | |
| **f. Availability of informants or other observers** | | | | | |  |
| **Answer Options** | | | | **Response Percent** | **Response Count** |  |
| 1 very important | | | | 14.7% | 5 |  |
| 2 | | | | 26.5% | 9 |  |
| 3 | | | | 26.5% | 9 |  |
| 4 | | | | 17.6% | 6 |  |
| 5 | | | | 2.9% | 1 |  |
| 6 | | | | 8.8% | 3 |  |
| 7 not at all important | | | | 2.9% | 1 |  |
| ***answered question*** | | | | | **34** |  |
| ***skipped question*** | | | | | **0** |  |

| **5. What is the maximum amount of time that a prescribing physician will reasonably spend assessing a patient for cognitive change with a procognitive treatment for schizophrenia?** | | |
| --- | --- | --- |
| **Answer Options** | **Response Percent** | **Response Count** |
| 1-2 minutes | 9.1% | 3 |
| 5 minutes | 66.7% | 22 |
| 15 minutes | 24.2% | 8 |
| 30 minutes | 0.0% | 0 |
| up to 60 minutes | 0.0% | 0 |
| ***answered question*** | | **33** |
| ***skipped question*** | | **1** |

| **15. Assuming that a procognitive medication demonstrated safety and efficacy, to what extent do you agree that it should be considered standard of care in patients with schizophrenia (1=strongly agree to 7=strongly disagree)?** | | |
| --- | --- | --- |
| **Answer Options** | **Response Percent** | **Response Count** |
| 1 strongly agree | 58.1% | 18 |
| 2 | 25.8% | 8 |
| 3 | 12.9% | 4 |
| 4 | 0.0% | 0 |
| 5 | 3.2% | 1 |
| 6 | 0.0% | 0 |
| 7 strongly disagree | 0.0% | 0 |
| ***answered question*** | | **31** |
| ***skipped question*** | | **3** |

Voting of audience participants that differed from panelists on key consensus questions

| **Informants (caregiver/family/employer) are vital to the assessment of response** | | |
| --- | --- | --- |
| **Answer Options** | **Response Percent** | **Response Count** |
| 1 Completely agree | 15.9% | 7 |
| 2 | 22.7% | 10 |
| 3 | 31.8% | 14 |
| 4 | 9.1% | 4 |
| 5 | 11.4% | 5 |
| 6 | 4.5% | 2 |
| 7 Completely disagree | 4.5% | 2 |
| ***answered question*** | | **44** |
| ***skipped question*** | | **1** |

| **Treatment of cognitive impairment in clinical practice should be initiated independent of a patient’s baseline level of everyday functioning.** | | |
| --- | --- | --- |
| **Answer Options** | **Response Percent** | **Response Count** |
| 1 Completely agree | 33.3% | 15 |
| 2 | 22.2% | 10 |
| 3 | 17.8% | 8 |
| 4 | 11.1% | 5 |
| 5 | 4.4% | 2 |
| 6 | 11.1% | 5 |
| 7 Completely disagree | 0.0% | 0 |
| ***answered question*** | | **45** |
| ***skipped question*** | | **0** |

References

1. Iverson GL, Brooks BL, Ashton VL, Johnson LG, Gualtieri CT. Does familiarity with computers affect computerized neuropsychologicl test performance? *J Clin Exp Neuropsychol.* 2009;31:594-604.
